# Use of remdesivir in the treatment of coronavirus disease 2019 (COVID-19) infection among Sudanese patients: a case series

**DOI:** 10.12688/f1000research.51375.1

**Published:** 2021-06-29

**Authors:** Maysoun Yousif, Ghada Abd El-Raheem, Doaa Mohamed

**Affiliations:** 1 Emergency, Imperial Hospital, Khartoum, Khartoum, 11114, Sudan; 2 Pharmacy, Imperial Hospital, Military Hospital, Soba University Hospital, Khartoum, Khartoum, 11114, Sudan; 3 Emergency Department, Imperial Hospital, Khartoum, Khartoum, 11114, Sudan

**Keywords:** COVID-19, treatment, liver enzymes, Remdesivir, hypotension, Imperial Hospital, Sudan, Sudanese patients.

## Abstract

**Introduction**: The coronavirus disease 2019 (COVID-19) pandemic is affecting populations worldwide. Remdesivir is an anti-retroviral agent, with a broad spectrum of usage. Remdesivir usage against COVID-19 had been studied both *in vitro* and *in vivo *but is still considered a new treatment for COVID-19 and is not available in all countries. The aim of our study was to report several cases of the use of remdesivir in Sudanese patients and report the adverse events related to the course of treatment.

**Methods:** A case series study was conducted in Imperial Hospital, Khartoum, Sudan reporting two cases who received remdesivir for treating COVID-19 besides other treatments such as steroids and supportive therapy in December 2020. Cases were males aged over 65 years.

**Cases presentation:** Both patients were severe cases of COVID-19 admitted to the intensive care unit (ICU), who received remdesivir for treating COVID-19 infection. Several side effects were reported: the first case had increased liver enzymes and then unexpectedly died from severe resistant hypotension; and hypoalbuminemia was noticed in the second case.

**Conclusions: **Remdesivir use among patients in Sudan must be studied extensively in order to determine the unexpected fatal event and assess the association of this event to remdesivir use, as well as to report the frequency of the side effects.

## Abbreviations

CPAP: Continuous Positive Airway Pressure

CT: Computed Tomography

DM: Diabetes Mellitus

FFP: Fresh Frozen Plasma

GCS: Glasgow Coma Scale

ICU: Intensive Care Unit

IgG: Immunoglobulin G

IgM: Immunoglobulin M

I.V.: Intravenously

MOH: Ministry of health

PCR: Polymerase Chain Reaction

RNA: Ribonucleic Acid

RT-PCR: Reverse Transcription Polymerase Chain Reaction

SARS-COV-2: Severe Acute Respiratory Syndrome

SPO _2_: Oxygen Saturation

## Introduction

The coronavirus disease 2019 (COVID-19) pandemic is affecting people worldwide with all populations susceptible to getting infected; especially health care personnel and elderly populations. ^1^ Concomitant comorbid conditions, such as diabetes mellitus (DM), are related to increased severity of COVID-19 symptoms. ^1, 2^ Remdesivir is an anti-retroviral agent, with a broad spectrum of activity. It is a prodrug, which was first developed to be used for the treatment of Ebola. ^3^ Recently, it was proposed to treat COVID-19 infection. ^4, 5^ The use of remdesivir against COVID-19 had been studied both *in vitro* and *in vivo,* but still considered new for COVID-19 treatment and not available in all countries. ^6, 7^ Although some remdesivir trials have not had enough power, ^8^ one large scale clinical trial has been done, in which, there was no statistically significant difference in the improvement time with the use of remdesivir (hazard ratio 1.23 [95% confidence interval (CI) 0.87–1.75]. Further, the course of remdesivir was stopped early due to the side effects noted in 12% of the patients which included increased alanine and other side effects. ^9^ The aim of our study was to report the use of remdesivir among two cases in Sudan and report the adverse events related to the course of treatment.

## Methods

A case series study design was implemented involving three Sudanese patients. Cases are described in detail below. The data were collected retrospectively from the medical records. Confidentiality of participants was assured through the use of an anonymous research tool that had no identifiers to the involved patients and documented the medical data through codes. This data collection sheet was developed by the researchers and validated by a specialist in research methodology with a PhD. Epi-info-7 was used.

### Ethical approval and participant consent

Approval was firstly obtained from the Medical administration of Imperial Hospital. The study proposal was then submitted to the Administration of Innovation and Scientific Research at the State Ministry of Health (MOH), Khartoum, Sudan. Expedited review was conducted by the IRB of the MOH and approval was granted. Another copy of the proposal was submitted to the Administration of Private Medical Facilities.

Since the study was conducted using the archived medical records of the patients after death or discharge, informed consent was obtained from surrogate decision makers by contacting them through their registered phone numbers. In our study, the wives gave the approval after consulting the family. The objectives of the study and what data would be collected from the patients' medical records was explained in full. The collected data were used strictly for the purpose of the study objectives.

## Case 1 presentation

A 78 years old male presented to the emergency isolation room at Imperial hospital, Khartoum, Sudan on 8 December 2020. The patient was tested for severe acute respiratory syndrome coronavirus 2 (SARS-CoV-2) infection and antibodies on 4 December 2020 (brand of tests unknown) and the results were received on 5 December. SARS-CoV-2 RNA by polymerase chain reaction (PCR) was detected, while the SARS-CoV-2 antibody test was negative. The patient had been complaining of shortness of breath for 3 days. His comorbidities were diabetes mellitus (DM) for which he was on insulin treatment, and a history of colon cancer for which he had an operation two 2 years previously and he was on oral chemotherapy. The patient had no other co-morbid conditions. A confirmatory test by using reverse transcription polymerase chain reaction (RT-PCR) was done on 10 December confirming the infection (brand of the test unknown). The medications in Table 1 were given to the patient from day 1 and planned for 10 days, besides 1.5 litres of fluids per day. The patient was admitted to the isolation ICU on the same day of hospital arrival.

**Table 1.** COVID-19 treatment medications administered to the patients.

**Medications**
Remdesivir 200 mg I.V. LD, 100 mg I.V. O.D.
Meropenem 1 gm I.V. B.D.
Enoxaparin 40mg S.C. B.D.
Dexamethasone 6 mg O.D. I.V.
Paracetamol 1gm I.V. on need
Pantoprazole 40 mg I.V. O.D.
Vitamin C 1000 mg O.D.
Zinc sulphate 15 mg O.D.
Azithromycin 500 mg O.D.
Salbutamol nebulized solution 6 Hourly
Ipratropium nebulized solution 6 Hourly
Pulmicort 0.5 gm nebulized solution 6 Hourly

COVID-19 = coronavirus disease 2019, LD = loading dose, I.V. = intravenously, O.D. = once daily, B.D. = twice daily.

Remdesivir was started on day 1, 8 December 2020. A loading dose of 200 mg was given in 250 mL of normal saline followed by a maintenance dose of 100 mg intravenously (I.V.) in 250 mL of normal saline once daily, planned for 10 days. On admission, the patient's oxygen saturation (SPO _2_) was 92% and continuous positive airway pressure (CPAP) was applied. Table 2 illustrates the daily vital signs of the patient.

**Table 2.** Daily vital signs of the patient (Case 1).

Vital signs	Day 1 8/12/2020	Day2 9/12l2020	Day 3 10/12/2020	Day 4 11/12/2020	Day 5 12/12/2020	Day 6 13/12/2020
**SPO**_**2**_	92%	94%	95%	94%	90%	83%, 80%
**RR**	30	35	33	30	32	34
**HR (taken twice/day day 1-3)**	100 bpm 109 bpm	128 115 bmp	122 130 bpm	130 bpm	132 bpm	125 bpm
**B.P.**	130/70 140/80	139/87 133/85	149/92 168/100	170/83	155/83	99/56 Undetectable 84/40
**GCS**	15/15	15/15	13/15	10/15	10/15	3/15
**RBG (taken twice/day)**	233 mg/dl 402 mg/dl	343 230	300 370	350 310	369 368	268 280
**Temp. (degrees Celsius)**	37.3	36.0	36.4			

SPO _2_ = oxygen saturation, RR = respiratory rate, HR = heart rate, B.P. = blood pressure, GCS = Glasgow Coma Scale, RBG = random blood glucose.

Laboratory examinations were done daily starting from day 3, 10 December 2020. The results are presented in Table 3.

**Table 3.** Results of laboratory examinations of the patient (Case 1).

**Laboratory tests**	**Day 3**	**Day 5**	**Laboratory tests**	**Day 3**	**Day 5**
**Blood Urea**	121 mg/dl		**pH**		7.347
**Serum creatinine**	1.2 mg/dl		**SO2%**		97.50%
**Na+ (Sodium)**	153 mmol/L	165.2 mmol/L	**HCO3**		27meq/L
**K+ (Potassium)**	5.3 mmol/L	4.66 mmol/L	**Hct**		49.50%
**D-dimer**	5mcg/ml		**Cl**		114.5 mmol/L
**CRP**	215 mg/L		**Total protein**	7.5 g/dL	7.6 g/dL
**TWBCs**	17.7*10^9/L		**Serum albumin**	2.4 g/dL	2.6 g/dL
**RBCs**	5.7*10^9/L		**ALP**	97 U/L	111 U/L
**Neutrophils**	88*10^9/L		**ALT (GPT)**	288 I.U/L	142 I.U/L
**Lymphocytes**	7*10^9/L		**AST (GOT)**	431 I.U/L	70 I.U/L
**PLts**	351*10^9/L		**Prothrombin time**	65 Sec	24 Sec
**PCO _2_**		50.6 mmHg	**INR**	>10	1.8
**PO _2_**		105.4 mmHg	**APTT**	49 Sec	

CRP = c-reactive protein, TWBCs = total white blood cell count, RBCs = red blood cells, PLts = platelets, PCO _2_ = partial pressure of carbon dioxide, PO _2_ = partial pressure of oxygen, SO _2_ = oxygen saturation, HCO _3_ = bicarbonate, Hct = hematocrit, Cl = chlorine, ALP = alkaline phosphatase, ALT (GPT) = alanine aminotransferase (glutamic pyruvic transaminase), AST (GOT) = aspartate aminotransferase (glutamic oxaloacetic transaminase), INR = international normalized ratio, APTT = activated partial thromboplastin time.

**Figure 1.**
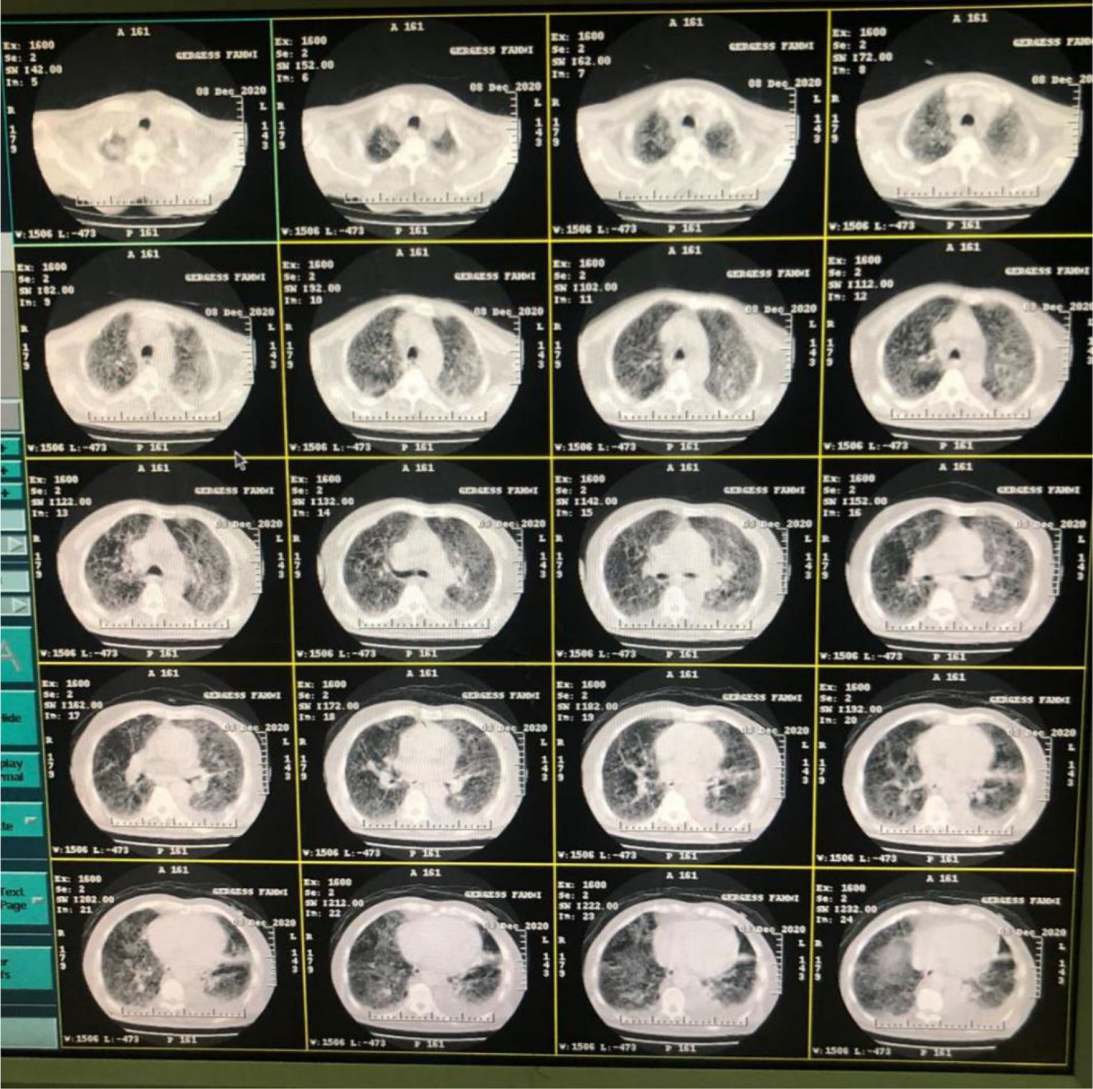
Chest computed tomography of case 1 on hospital arrival.

Chest computed tomography was done for the patient on the first day of hospital arrival, 8 December, shown in Figure 1.

On day 3, the patient started deteriorating as the GCS was reducing. Nasogastric feeding was started and I.V. fluids were increased to 3 litres per day alongside the same plan. On day 6, severe hypotension had occurred in the patient, to an extent that blood pressure was undetectable on any monitor or manual devices. Hypotension was resistant to fluids and vasopressors which eventually led to the death of the patient. The patient was deceased on day 6, 13 December 2020, as a result of severe refractory hypotension.

## Case 2 presentation

A 75 years old male presented to the Emergency Isolation room at Imperial hospital on 18 December 2020, with generalized fatigue and fever for 1 week, and hemiparesis. Co-morbid conditions of the patient were a pacemaker device and benign prostate enlargement (BPH) condition, which was operated on five years previously. Oxygen saturation with CPAP mask was 87%. The patient was diagnosed with COVID-19, pneumonia and sepsis which were confirmed by PCR and CT-chest. An incidence of haemorrhagic stroke was confirmed through brain imaging. The patient was admitted to the Isolation ICU immediately, with reduced GCS (9/15). Respiratory support was initiated with physiotherapy and prone positioning for 16 hours per day. The patient was administered 3 units of fresh frozen plasma (FFP) once, platelet concentrate 50 U/Kg once and vitamin K 10 mg I.V. once. The patient developed hypernatremia (Serum Na ^+^ 153), International normalized ratio (2.8). Remdesivir was initiated on day 1 of admission (18 December 2020), by the dose detailed in Table 1, and stopped on day 5, 23 December 2020. Hypoalbuminemia was noticed on 24 December (2.2 g/dl), for which albumin 20% infusion was given twice daily for 4 days, the serum albumin level results after treatment were not recorded. On 27 December, the 10 ^th^ day of hospital admission, the patient was transferred to a different healthcare facility, based on the request of the surrogate decision maker of the patient, and we had no follow up information after the discharge from Imperial Hospital. The medications in Table 1, were given exactly to the patient during the hospital stay, same as for Case 1.

## Discussion

Our patients were aged 75 and 78 years, similar ages to other published studies ^9, 10^ being above 65 years. Both study cases were male, as COVID-19 infection is more prevalent in males. ^10‐ 12^ In our study, case 1 was diabetic, which is known to be associated with increased COVID-19 severity of infection. ^2^ This comorbidity was reported for 25% and 16% of patients who received remdesivir. ^9, 10^ Remdesivir is considered a viable treatment option in severe COVID-19 infections. ^13, 14^ Remdesivir doses administered for our patients were a 200 mg intravenously loading dose followed by 100 mg daily dose, which is the recommended dosage. ^9, 15^ Both cases had received antibiotics and steroids along with remdesivir, which is in line with. ^9^

Hypoalbuminemia has been reported in patients taking remdesivir, ^9^ as in case 2 of our study. But case 1 had hypoalbminemia on presentation. The case 1 patient had 6 treatment days with remdesivir, with no improvement, in contrast to published trials in which remdesivir treatment course of 5 days has shown significant improvements. ^16, 17^ Our second case patient received remdesivir after 4 days of presentation of COVID-19 symptoms, which is consistent with published literature reporting that the efficacy of remdesivir is higher in patients who receive it within 10 days of symptom presentation. ^18^ While another study concluded that delayed treatment initiation with remdesivir was beneficial as well. ^19^

Liver enzymes abnormalities have also been reported among patients who received remdesivir. ^4, 10, 20^ For such cases, treatment course should be stopped. ^14^ In our first case, liver enzymes were extremely elevated since day 3 of treatment ( Table 3), but remdesivir course had not been discontinued. One of our cases was deceased and that was expected, as per a case series study in US, in which 50% of patients with severe COVID-19 infection on remdesivir were deceased. ^21^ Also, in a clinical study only 3% of patients on remdesivir had clinical improvement on day 7. ^9^ In another study, compassionate use of remdesivir resulted in improvements in 69% of patients. ^10^ The cause of death was severe resistant hypotension, which has been reported as a serious event in remdesivir use. ^10, 20^

The limitations of our study were the observational nature of case series that cannot establish direct cause-effect relationships. Also, the data were collected retrospectively from the medical records of the patients which had some missing medical results.

## Conclusions and recommendations

Both patients were severe cases of COVID-19 admitted to the ICU. Unexpectedly, severe resistant hypotension was the cause of death of one of the patients. Increased liver enzymes was noticed in one case (case 1), as well as hypoalbuminemia (case 2). Wider studies regarding remdesivir use among patients in Sudan must be conducted extensively in order to study this unexpected fatal event and assess the association of this event to remdesivir use, as well as, to report the frequency of the side effects.

## Consent

Written informed consent for publication of their clinical details and images was obtained from the surrogate decision makers of the patients. The surrogate decision makers gave written informed consent before data was collected and this was confirmed again after the manuscript was completed.

## Data availability

All data underlying the results are available as part of the article and no additional source data are required.
