# Supplementary material for: Use of Remdesivir in the treatment of Coronavirus Disease 2019 (COVID-19) infection among Sudanese patients: a case series
Source: F1000Res. 2022 Mar 10;10:512. Originally published 2021 Jun 29. [Version 2] doi: 10.12688/f1000research.51375.2 (PMC8941288; doi:10.12688/f1000research.51375.2)
Supplement: Peer review history

# Peer review: Reviewer response for version 2

**DOI:** 10.5256/f1000research.122150.r126872

**Published:** 2022-03-22

**Authors:** Andri Frediansyah

**Affiliations:** 1 National Research and Innovation Agency (BRIN), Jakarta, Indonesia; 2 Indonesian Institute of Sciences (LIPI), Wonosari, Indonesia

I am satisfied with the corrections made by the authors.

Are enough details provided of any physical examination and diagnostic tests, treatment given and outcomes?

Partly

Is the background of the cases’ history and progression described in sufficient detail?

Yes

Is sufficient discussion included of the importance of the findings and their relevance to future understanding of disease processes, diagnosis or treatment?

Yes

Is the conclusion balanced and justified on the basis of the findings?

Yes

Reviewer Expertise:

Pharmaco Biology

I confirm that I have read this submission and believe that I have an appropriate level of expertise to confirm that it is of an acceptable scientific standard.

# Peer review: Reviewer response for version 1

**DOI:** 10.5256/f1000research.54536.r122200

**Published:** 2022-02-23

**Authors:** Andri Frediansyah

**Affiliations:** 1 National Research and Innovation Agency (BRIN), Jakarta, Indonesia; 2 Indonesian Institute of Sciences (LIPI), Wonosari, Indonesia

Two COVID-19 cases (aged 74 and 75 years) were treated at Sudan's Imperial Hospital, and the authors conducted a case series study to assess remdesivir's efficacy. According to two case series, adverse events such as elevated liver enzymes and hypoalbuminemia were observed in patients with severe COVID-19 infection who were admitted to the intensive care unit. Due to the limited number of case studies in this study, conclusions about remdesivir's efficacy in the general population are impossible. However, the case series report is interesting and well-presented, making it worth reading during this period of emergencies. The following points, however, should be consider:

- Please write IRB in full name with no abbreviation.
- Please include the number of the ethical approval.
- Kindly explain Figure 1 in the paragraph that follows.
- Is there any laboratory or computed tomography data available in case 2? Is it still possible to present if the response is negative?

Are enough details provided of any physical examination and diagnostic tests, treatment given and outcomes?

Partly

Is the background of the cases’ history and progression described in sufficient detail?

Yes

Is sufficient discussion included of the importance of the findings and their relevance to future understanding of disease processes, diagnosis or treatment?

Yes

Is the conclusion balanced and justified on the basis of the findings?

Yes

Reviewer Expertise:

Pharmaco Biology

I confirm that I have read this submission and believe that I have an appropriate level of expertise to confirm that it is of an acceptable scientific standard, however I have significant reservations, as outlined above.

# Response

**Published:** 2022-03-04

**Authors:** Ghada Abd El-Raheem

**Affiliations:** Imperial Hospital, Soba University Hospital, Military Hospital, Sudan

Dear Reviewer,

 Thank you for your valuable comments. I have fulfilled them all.

 Thank you again for your precious time.

# Peer review: Reviewer response for version 1

**DOI:** 10.5256/f1000research.54536.r99911

**Published:** 2021-12-03

**Authors:** Bashir Alsiddig Yousef

**Affiliations:** 1 Department of Pharmacology, Faculty of Pharmacy, University of Khartoum, Khartoum, Sudan

Authors performed a case series study to assess the use of remdesivir among two COVID-19 cases in Imperial Hospital, Khartoum, Sudan. Generally, the study is interesting and well presented. However, some points which should be reconsidered in this study:

- As the study focused on the adverse events related to the use of remdesivir, I think the authors need to change the title to highlight the adverse events of remdesivir.
- Is it conducted in two or three patients? Please correct the number in the methodology section.
- Authors need to cite the guidelines for using the medications for treating COVID-19 cases.

Are enough details provided of any physical examination and diagnostic tests, treatment given and outcomes?

Yes

Is the background of the cases’ history and progression described in sufficient detail?

Yes

Is sufficient discussion included of the importance of the findings and their relevance to future understanding of disease processes, diagnosis or treatment?

Partly

Is the conclusion balanced and justified on the basis of the findings?

Yes

Reviewer Expertise:

Pharmacology, Clinical Pharmacy, Clinical Pharmacology

I confirm that I have read this submission and believe that I have an appropriate level of expertise to confirm that it is of an acceptable scientific standard.

# Response

**Published:** 2022-03-04

**Authors:** Ghada Abd El-Raheem

**Affiliations:** Imperial Hospital, Soba University Hospital, Military Hospital, Sudan

Dear Reviewer,

 Thank you for your valuable comments. I have fulfilled them all in the updated version.

 Thank you again for your precious time.
